# Sound Event Localization and Detection Using Imbalanced Real and Synthetic Data via Multi-Generator

**DOI:** 10.3390/s23073398

**Published:** 2023-03-23

**Authors:** Yeongseo Shin, Chanjun Chun

**Affiliations:** Department of Computer Engineering, Chosun University, Gwangju 61452, Republic of Korea; ys070400@chosun.ac.kr

**Keywords:** sound event detection, sound localization, residual convolutional neural network, transformer

## Abstract

This study proposes a sound event localization and detection (SELD) method using imbalanced real and synthetic data via a multi-generator. The proposed method is based on a residual convolutional neural network (RCNN) and a transformer encoder for real spatial sound scenes. SELD aims to classify the sound event, detect the onset and offset of the classified event, and estimate the direction of the sound event. In Detection and Classification of Acoustic Scenes and Events (DCASE) 2022 Task 3, SELD is performed with a few real spatial sound scene data and a relatively large number of synthetic data. When a model is trained using imbalanced data, it can proceed by focusing only on a larger number of data. Thus, a multi-generator that samples real and synthetic data at a specific rate in one batch is proposed to prevent this problem. We applied the data augmentation technique SpecAugment and used time-frequency masking to the dataset. Furthermore, we propose a neural network architecture to apply the RCNN and transformer encoder. Several models were trained with various structures and hyperparameters, and several ensemble models were obtained by “cherry-picking” specific models. Based on the experiment, the single model of the proposed method and the model applied with the ensemble exhibited improved performance compared with the baseline model.

## 1. Introduction

SELD is commonly performed to preprocess audio signals. SELD can be divided into subtasks called sound event detection (SED) and sound source localization (SSL).

SED aims to classify a specific sound event in a sound [1] and detect the onset and offset of each sound event [2,3]. In real life, situations such as background noise, overlapping sound sources, and the occurrence of sound sources belonging to the same event class can occur. Overlapping sources refer to situations where multiple event classes occur simultaneously in a given time instance, which can be addressed by applying polyphonic SED that can detect multiple overlapping event classes at the same time. Additionally, there are scene-dependent and scene-independent approaches in SED. Scene-dependent approaches provide information on the acoustic scene for both training and testing, while scene-independent approaches do not provide such information. Such SED tasks can be utilized in various fields in which sound is detected, such as surveillance [4] or audio captioning [5].

SSL is the task of estimating the location of a sound source in three-dimensional coordinates using the recorded multichannel audio signal, with the microphone being the reference point for the recording. Like the human auditory system, which utilizes physical variables such as time, intensity, and spectral shape to perform SSL, deep learning-based SSL also estimates these variables, such as time, intensity, and spectrogram, to localize sound sources. SSL is not only a task of estimating direction, but there are also tasks that consider distance as well. In this study, SSL simplifies the direction-of-arrival (DOA) estimation by only considering azimuth and elevation without estimating the distance to the microphone array. Such SSL is used in various fields such as source separation [6], speech recognition [7], and speech enhancement [8]. In SELD, two subtasks, SED and SSL, are performed simultaneously.

There is a challenge called DCASE that performs SELD. The DCASE challenge was first held in 2013 and has continued until 2022. The DCASE Task 3 challenge on SELD was held in 2019, and various deep-learning-based methods have been proposed. Yearly, participants in the Task 3 challenge attempt to achieve the SELD in various environments. DCASE 2019 aimed to perform SELD using audio data containing only static sound sources. DCASE 2020 was similar to that of 2019; however, a dynamic sound source was added [9]. In 2021, directional interference was added to the 2020 audio data [10].

As the challenge progressed, numerous deep learning-based methods for improving SELD performance were proposed. The proposed method differentiates input features depending on the type of audio data format used, such as multichannel microphone array (MIC) and first-order ambisonic (FOA). Both formats commonly use mel-energy, but different approaches have been proposed for each format: intensity vectors(IV) for encoding time-difference characteristics in FOA format, and the use of Spatial Cue-Augmented Log-Spectrogram (SALSA) [11] and SALSA-Lite [12] for MIC format. SALSA employs a linear-frequency log-power spectrogram for each channel and an eigenvector-based phase vector (EPV). On the other hand, SALSA-Lite uses a modified input feature that replaces EPV with simple frequency-normalized inter-channel phase difference (NIPD). SALSA-Lite is more computationally efficient than SALSA. Furthermore, activity-coupled Cartesian direction-of-arrival (ACCDOA) [13] and Multi-ACCDOA [14] have been proposed as output formats for the SELD models. A neural-network-based SELD generally comprises a two-branch [9,15] and two-stage [16] method. The two-branch method estimates SED and DoA using two branches composed of fully connected layers in a single network. This approach linearly combines the weights and losses of the model for multi-objectives. However, the model’s performance can be significantly affected by the weight and might be difficult to tune. The two-stage method uses two separate networks to estimate SED and DoA. This approach avoids the multi-objective problem because it builds a network for each target. However, it increases the complexity of the system and the overall size of the network. A method proposed to address the issues of the two methods is ACCDOA. ACCDOA uses a single network and a single branch by assigning the sound event activity to the length of the corresponding Cartesian DoA vector. However, since ACCDOA is a class-wise output format, overlapping events cannot be detected in the same class. This problem was solved using multi-ACCDOA, which extended track-wise. The multi-ACCDOA format can detect overlapping sounds in the same class, resolving the problem of ACCDOA.

DCASE 2019–2021 were conducted using the synthesized data. However, DCASE 2022 Task 3 differs from previous competitions in that it includes a relatively small amount of real spatial acoustic scene data and a relatively large amount of synthetic data generated using specific indoor impulse responses [17]. There is a difference with the dataset. Furthermore, since the final evaluation uses only real data, the model’s performance is highly dependent on how well it generalizes when trained using large amounts of synthetic data. Therefore, in order to achieve optimal performance, participants need to solve the challenge of training a model with a disproportionate amount of data appropriately.

The two methods discussed subsequently are suggested for effective training, using imbalanced real and synthetic data. First, an RCNN and transformer encoder [18]-based neural network architecture are proposed. Furthermore, a multi-generator method is introduced that samples actual and synthetic data at a predetermined rate in a single batch. For the performance of the proposed SELD model, the error rate and F1-score are used as evaluation metrics for SED using the DCASE 2022 dataset, and the class-dependent localization error and recall are used as evaluation metrics for SSL [19]. The performance of the proposed SELD model is then compared with that of the DCASE 2022 baseline model.

Various proposed techniques were applied in the DCASE 2022 Task3 baseline. The model uses 7-channel data as input. In the FOA format, 4-channel mel-spectrogram and 3-channel IV are used, while in the MIC format, 4-channel mel-spectrogram and 3-channel SALSA-Lite are used. In addition, the baseline model consists of three layers of CNN with a kernel size of 3×3, two layers of Bi-GRU, and two layers of the FC layer.

The remainder of this paper is organized as follows. Section 2 describes the structure and method of the proposed model. Section 3 compares and evaluates the performances of the models. Finally, the conclusions are provided in Section 4.

## 2. Proposed Sound Event Detection and Localization Method

### 2.1. Architecture

**Figure 1:**
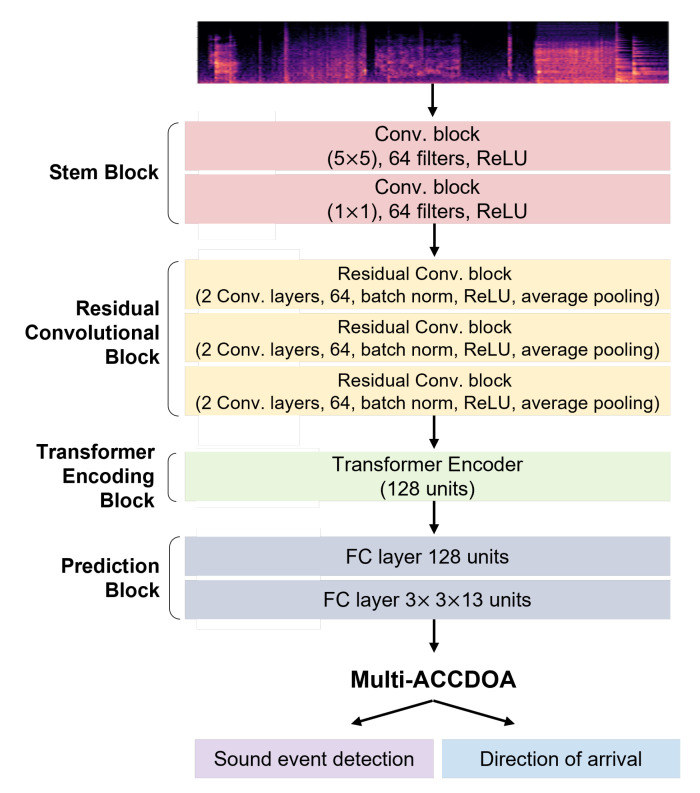
Architecture of the proposed RCNN and transformer-based model.

**Figure 2:**
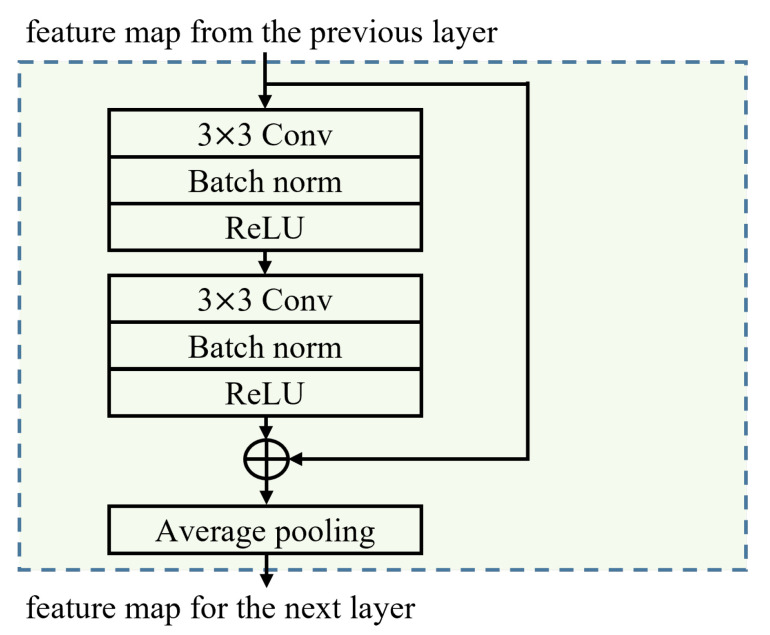
Block diagram of a residual convolutional block comprising a CNN layer with residual connections, batch normalization, and max pooling layer.

The structure of the proposed model is shown in Figure 1. The proposed neural network model is based on the RCNN structure. RCNN is an advanced form of residual network (ResNet) that uses residual blocks, which have the same input and output dimensions and add the input values to the output values, to minimize information loss [20]. In our proposed method, the stem block is formed using convolutional blocks to utilize the RCNN structure of the model. Here, the kernel sizes of the convolution block are 5 × 5 and 1 × 1. The residual block comprises a convolutional neural network (CNN) layer with residual connections, batch normalization, and a max pooling layer. The structure of the residual block is shown in Figure 2. The CNN with residual blocks utilized a kernel size of 3×3, and a total of three layers of residual blocks were used. Then, the transformer encoder and fully connected layers are formed. The hyper tangent is used as an activation function. The transformer encoder is a sequence model that preserves the positional information of input data and can simultaneously learn all positional relationships in the input sequence. Unlike recurrent neural network (RNN), it does not require sequential processing, which makes it faster, and can achieve better performance than RNN when the input sequence is long [18]. The attention model used in the transformer encoder of the proposed model employs multi-head attention. For the hyperparameters, we set the encoder dimension to 128, the number of layers to 1, the number of heads to 4, and the dimension of the feedforward layer to 256, 512, 1024, and 2048. The hyper tangent is used as an activation function. Finally, a single model with a single branch is trained and Multi-ACCDOA is applied as the final output format since SELD is performed for overlapping sources. The format is defined as follows:

(1) $$P\in R^{3\times N\times C\times T},$$

where *N*, *C*, and *T* denote the track, class, and frame index, respectively.

We used the FOA format of four channels as the audio data format. Four mel-spectrograms extracted from each channel and the IV of three channels were used as input features of the model. The extracted mel-spectrograms and IVs were stacked to form a total of seven input channels. The IV is used to estimate the DOA in FOA B-format recordings [21]. The FOA B-format comprises four channels and can be expressed as *W*, *X*, *Y*, and *Z*. IV can be calculated using the four-channel spectrogram that is the short-time Fourier transform output [22].

(2) $$I\left[t,k\right]=R\left(W*,h\left[t,k\right]\right)={\left[I_{X}\left[t,k\right],I_{Y}\left[t,k\right],I_{Z}\left[t,k\right]\right]}^{T},$$

where $h\left[t,k\right]={\left[X\left[t,k\right],Y\left[t,k\right],Z\left[t,k\right]\right]}^{T}$. R(·) represents the real part of the complex number, and (·)* represents the complex conjugate number. The IVs are calculated for the *X*, *Y*, and *Z* channels based on the w channel in the mel-spectrogram of the four channels.

### 2.2. Multi-Generator

The DCASE dataset consisted of 1200 synthetic data and 121 real data. According to the DCASE regulations, additional synthetic data were obtained, and a dataset consisting of 6000 synthetic data and 121 real data was complied with. Among the synthetic data, 5700 were used for training, and the real dataset was composed of 67 examples designated by DCASE. The ratio of real and synthetic data in the given dataset is imbalanced. Owing to the limited number of real data, a multi-generator was presented to utilize the data efficiently for training. The generator plays a role in loading data for model training and evaluation. When an imbalanced dataset is used for training, only a relatively large amount of data can be focused on. Thus, we utilized the multi-generator to prevent this scenario. The multi-generator distinguishes generators according to the type of data. Further, it allows for sampling of real and synthetic data according to the predetermined ratio. Accordingly, it does not focus only on one data type. Here, the batch size was set to 128. We used two generators, one for real data and one for synthetic data, to sample a batch consisting of 8 real data and 120 synthetic data.

The DCASE dataset consisted of 1200 synthetic data and 121 real data. We complied with the competition regulations and acquired additional synthetic data, resulting in the construction of a dataset consisting of 6000 synthetic data and 121 real data. Among these, 6000 synthetic data and 67 real data were used for training. For validation data, we used the real data separated by DCASE. The ratio of real and synthetic data in the given dataset is imbalanced, which leads to an imbalance in the ratio of the data used for training. Owing to the limited number of real data, a multi-generator was presented to utilize the data efficiently for training. The generator performs a role same as that of a dataloader, loading the dataset. A single generator was used to train the model in the existing training method. However, when an imbalanced dataset is used for training, only a relatively large amount of data can be focused on. Thus, we utilized the multi-generator to prevent this scenario. Multi-generator regularly samples synthetic and real data at a predetermined rate. Accordingly, it does not focus only on one data type. Here, the batch size was set to 128. We used two generators, one for real data and one for synthetic data, to sample a batch consisting of 8 real data and 120 synthetic data.

The dataset used in this study consists of 6000 synthetic data and 121 real data. Among the synthetic data, 5700 were used for training, and the real dataset was composed of 67 examples designated by DCASE. The ratio of real and synthetic data in the given dataset is imbalanced. Owing to the limited number of real data, a multi-generator was presented to utilize the data efficiently for training. The generator plays a role in loading data for model training and evaluation. When an imbalanced dataset is used for training, only a relatively large amount of data can be focused on. Thus, we utilized the multi-generator to prevent this scenario. The multi-generator distinguishes generators according to the type of data. In addition, it allows for sampling of real and synthetic data according to the predetermined ratio. Accordingly, it does not focus only on one data type. Here, the batch size was set to 128. We used two generators, one for real data and one for synthetic data, to sample a batch consisting of 8 real data and 120 synthetic data.

### 2.3. Training Method

The hyperparameters to train the model are as follows: Nesterov momentum Adam [23] was employed as the optimizer. The dropout and learning rates were set to 0.2 and $10^{-3}$, respectively. The learning rate scheduler was also employed for training. Particularly, we trained the neural network model with the same learning rate until 30 epochs. Subsequently, cosine annealing scheduling was utilized [24]. This starts with a relatively large learning rate and is dropped rapidly to the predefined minimum value. Here, the maximum and minimum learning rates were $10^{-3}$ and $10^{-4}$, respectively.

**Figure 3:**
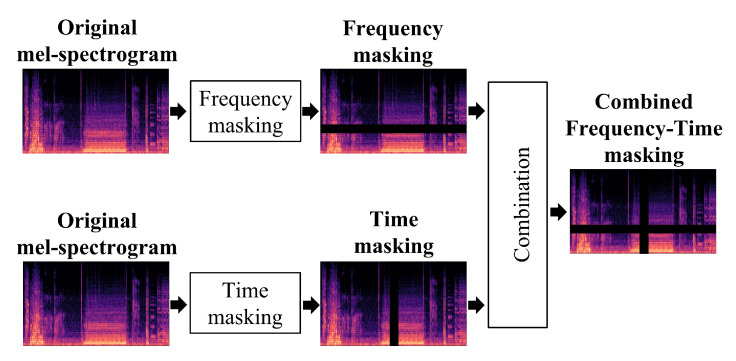
Example of time and frequency masking.

Furthermore, the SpecAugment data augmentation method, widely used in speech processing, was employed [25]. Two types of masking were applied in SpecAugment: frequency and time masking. In frequency masking, consecutive frequencies of a spectrogram are randomly masked. Time masking is similar to frequency masking; however, consecutive time frames of a spectrogram are randomly masked. The spectrogram with two masking applied is shown in Figure 3. SpecAugment was randomly applied to each batch of input data. Time masking could be applied to the entire sequence, with a width of 8 frames, masking roughly 0.02 s of time information per batch. Frequency masking is also applied to the entire sequence, with 8 mel-bins masked out of a total of 64 mel-bins.

## 3. Performance Evaluations

Model performance was measured only on a limited number of real data in the development dataset. In addition, it was evaluated according to the SELD evaluation metrics used in the DCASE challenge; error rate, F1-score, localization error, and localization recall [19]. Here, error rate and F1-score were utilized as evaluation metrics for SED, and localization error (${LE}_{CD}$) and localization recall (${LR}_{CD}$) were used as DOA evaluation metrics, respectively. The error rate was determined using the number of errors in terms of insertions (*I*), deletions (*D*), substitutions (*S*), and reference events (*N*), and it is represented as

(3) $$ER=\frac{D+I+S}{N}.$$

The F1-score was calculated over all test data based on the total number of false positives (*FP*), false negatives (*FN*), and true positives (*TP*). The F1-score is expressed as

(4) $$P=\frac{TP}{TP+FP},\;R=\frac{TP}{TP+FN},\;F=\frac{2PR}{P+R},$$

where *P* and *R* are the mean precision and recall, respectively.

Class-dependent localization metrics are calculated based on class-aware localization. The class-aware localizations ${LE}_{C}$ and ${LR}_{C}$ are computed only between predictions and references of class *c*. ${LE}_{C}$ and ${LR}_{C}$ are defined as

(5) $${LE}_{c}=\frac{{\left|\right|A_{c}⨀D_{c}\left|\right|}_{1}}{{\left|\right|A_{c}\left|\right|}_{1}},$$

(6) $${LR}_{c}=\frac{\sum_{l}{\left|\right|A_{c}^{\left(l\right)}\left|\right|}_{1}}{\sum_{l}\;N_{c}^{\left(l\right)}},$$

where $A_{c}$, $D_{c}$, and $N_{c}$ indicate the association matrix, distance matrix, and references, respectively. $c\in \left[1,\ldots ,C\right]$ represents the class index, and l=1,…,L represents temporal frame outputs or some other preferred averaged segmental representation. Using the calculated $LE_{c}$ and $LR_{c}$, the overall class-dependent $LE_{CD}$ and $LR_{CD}$ are determined as the class means as follows

(7) $${LE}_{CD}=\frac{1}{C\cdot L}\sum_{c}\sum_{l}{LE}_{c}^{\left(l\right)},$$

(8) $${LR}_{CD}=\frac{1}{C}\sum_{c}{LR}_{c}.$$

The SELD score is not included in the ranking calculation, but it is used as a performance metric to assess overall performance during model training. The SELD score is calculated by averaging four metrics: error rate, F1-score, localization error, and localization recall. The SELD score is calculated as follows:

(9) $$SELDscore=\frac{Errorrate+(1-F1)+LE+LR}{4}.$$

The resulting value ranges from 0 to 1, with better model performance resulting in values closer to 0.

The performance between models follows the DCASE ranking system. Sort in ascending order based on the cumulative rank of the four metrics. For example, if model A is ranked as error rate: 1, F1-score: 1, localization error: 3, localization recall: 1, then the cumulative rank is 1 + 1 + 3 + 1 = 5. If the individual ranks of model B are error rate: 2, F1-score: 3, localization error: 1, localization recall: 2, the cumulative rank is 8. As a result, the overall ranking is A, B.

Performance was compared using a validation set composed only of real data, and the training was assumed to converge if there was no improvement in performance after 100 epochs. Table 1 shows the model’s performance for the training method as an evaluation metric. The proposed three training methods differ in the types of data used. Method 1 used only synthesized data and Methods 2–3 used the same synthesized data and real data but were trained with different approaches. Methods 1–3 were evaluated with real data to calculate performance metrics. Method 2 was fine-tuned with real data after training with synthetic data. Method 3 trained the model using multi-generators, as the proposed method in this paper. Such models were constructed with the same neural network architecture. Comparing method 1 and method 2, using real data for training showed better performance in all metrics. This indicates that there are differences between synthetic and real data. Therefore, using a small amount of real data appropriately in training is an important factor in improving performance. When comparing Method 2 and Method 3, the fine-tuning model received a better score in error rate (0.63), while the model with a multi-generator received better scores in the other metrics (F1: 0.31, LE: 23.17, LR: 62.0). As DCASE uses four evaluation metrics, the multi-generator, which showed overall improvement in three metrics, was deemed appropriate to use.

We trained various models by modifying hyperparameters based on models that applied RCNN and transformer encoder or bi-directional long-short-term memory (Bi-LSTM) to the RNN layer. We trained various types of models by changing the hyperparameters, such as the dimension of the transformer encoder. Specifically, we set the dimension to 256, 512, 1024, and 2048 and trained the models accordingly. Additionally, the L1 loss function or mean squared error (MSE) loss function was applied as a loss function. We cherry-picked the top 7 models with the best performance among the various models trained and presented the results in Table 2. The model using Bi-LSTM achieved high in terms of error rate (0.65) and localization error (22.24). In terms of error rate (0.65), F1-score (0.33), and localization recall (62.0), it was confirmed that the model with a transformer encoder showed the excellent performance. Out of all seven models, six models have transformer encoders constructed. In other words, the transformer encoder model outscored overall in all metrics. Accordingly, Model 7 with a 512-dimensional transformer encoder and L1 loss function outperforms compared to the other models by cumulative ranking.

The ensemble technique [26] was also applied to the model shown in Table 2. Particularly, the ensemble was applied to seven models in numerous forms. After that, the four ensemble models listed in Table 3 are lastly determined by cumulative ranking. Model I is the result of an ensemble of seven models, and Models II to IV are the results of an ensemble of six models in different combinations. Model II was ensemble of six models listed in Table 2, which were selected as 1, 3, 4, 5, 6, and 7. Model III was an ensemble of six models, which were selected as 1, 2, 4, 5, 6, and 7. Model IV was an ensemble of six models, which were selected as 1, 2, 3, 4, 6, and 7. All four ensemble models were conducted similarly in terms of error rate (0.59). Model I scored the highest in F1-score (0.35) and localization error (20.7), and Model IV showed the best in localization recall (59.0). In the cumulative ranking, the ensemble model, Model I, performed better than the other ensemble models.

The final performance results for the validation and evaluation dataset are depicted in Table 4. The proposed method models (Single Model 7, Ensemble Model 3) were based on the highest score in the cumulative ranking. In the case of the single model, the Model 7 received the highest cumulative ranked shown in Table 2. In the case of the ensemble model, Model III had the highest cumulative rank shown in the DCASE challenge result. Note that the evaluation dataset was not provided to participants, and model outcomes were measured by DCASE. Therefore, there is no performance evaluation for the proposed method on the eval set that was not submitted. On the validation data, the ensemble model performed as well on error rate (0.59) and F1-score (0.35), whereas the localization error (26.41) and localization recall (62.0) were better handled by the single model. On the evaluation data, the baseline model scored higher in localization recall (51.4). The proposed methods showed overall better performance on the Dev set compared to the baseline model. In addition, it was confirmed that the proposed method improved all three performance metrics except for Localization Recall on the Eval set. Since SELD evaluates based on four performance metrics, the proposed method that showed performance improvement in three metrics can be considered significant. Moreover, the proposed method achieved higher ranks than the baseline model in the cumulative ranking [27]. Consequently, it is implied that the proposed method appears to be better than the baseline and benefits SELD tasks.

## 4. Conclusions

In this paper, the sound event localization and detection method using the RCNN and transformer encoder was proposed for real spatial sound scenes. We made efforts to construct stem blocks and minimize the vanishing gradient by utilizing residual blocks. Additionally, we enhanced the model structure by replacing the conventional RNN with transformer encoders. To address the issue of the imbalanced dataset, we applied a multi-generator approach. This approach allows for proper sampling of real and synthetic data according to the defined ratio. Moreover, we applied SpecAugment as a data augmentation technique. We evaluated the proposed approach using performance metrics used in SELD and observed improved performance compared to the baseline model. In future research, we aim to advance the proposed approach by applying additional data preprocessing and deep learning techniques to further improve the overall performance.

## Figures and Tables

**Table 1:** Comparison of the trained model performances depending on the training methods. The values that have the best performance are bolded.

Method	Validation (Only Real Data)				
	Error Rate	F1-Score	*LE*	*LR*	SELD Score
Method 1 (training with synthetic data)	0.81	0.19	39.94	53.0	0.578
Method 2 (training with synthetic data, fine tuning with real data)	**0.63**	0.27	39.49	55.0	0.507
Method 3 (using multi-generator)	0.70	**0.31**	**23.17**	**62.0**	**0.475**

**Table 2:** Performance comparison of the trained single models. The values that have the best performance are bolded.

Method	Validation (Only Real Data)				
	Error Rate	F1-Score	*LE*	*LR*	SELD Score
Model 1 (Transformer encoder, 512 dim, MSE)	0.67	**0.33**	24.63	61.0	0.467
Model 2 (Transformer encoder, 2048 dim, L1)	0.69	0.30	24.03	58.0	0.486
Model 3 (Transformer encoder, 512 dim, L1)	**0.65**	0.31	24.81	59.0	0.472
Model 4 (Model 1 additional training)	0.66	0.30	25.72	**62.0**	0.471
Model 5 (Bi-LSTM, MSE)	**0.65**	0.31	**22.24**	53.0	0.483
Model 6 (Transformer encoder, 256 dim, MSE)	**0.65**	0.31	30.01	59.0	0.479
Model 7 (Model 3 additional training)	**0.65**	0.31	26.41	**62.0**	**0.467**

**Table 3:** Performance comparison of the ensemble models. The values that have the best performance are bolded.

Ensemble Model	Validation (Only Real Data)				
	Error Rate	F1-Score	*LE*	*LR*	SELD Score
Model I	**0.59**	**0.35**	**20.7**	57.0	**0.446**
Model II	**0.59**	0.34	24.8	58.0	0.452
Model III	**0.59**	**0.35**	33.8	57.0	0.464
Model IV	**0.59**	0.34	23.0	**59.0**	0.447

**Table 4:** Comparison of the performance of the proposed method with that of the baseline model. The values that have the best performance are bolded.

Challenge Result	Dev set					Eval Set				
	Error Rate	F1-Score	LE	LR	SELD Score	Error Rate	F1-Score	LE	LR	SELD Score
Baseline of DCASE 2022	0.71	0.21	29.3	46.0	0.551	0.61	0.24	22.9	**51.4**	0.496
Prposed method (Single Model 7)	0.65	0.31	**26.41**	**62.0**	0.467	-	-	-	-	-
Prposed method (Ensemble, Model III)	**0.59**	**0.35**	33.8	57.0	**0.464**	**0.59**	**0.31**	**19.8**	50.7	**0.471**

## Data Availability

Not applicable.
